# Human Pathogen Shown to Cause Disease in the Threatened Eklhorn Coral *Acropora palmata*

**DOI:** 10.1371/journal.pone.0023468

**Published:** 2011-08-17

**Authors:** Kathryn Patterson Sutherland, Sameera Shaban, Jessica L. Joyner, James W. Porter, Erin K. Lipp

**Affiliations:** 1 Department of Biology, Rollins College, Winter Park, Florida, United States of America; 2 Odum School of Ecology and Department of Environmental Health Science, University of Georgia, Athens, Georgia, United States of America; 3 Odum School of Ecology, University of Georgia, Athens, Georgia, United States of America; 4 Department of Environmental Health Science, University of Georgia, Athens, Georgia, United States of America; Northeastern University, United States of America

## Abstract

Coral reefs are in severe decline. Infections by the human pathogen *Serratia marcescens* have contributed to precipitous losses in the common Caribbean elkhorn coral, *Acropora palmata*, culminating in its listing under the United States Endangered Species Act. During a 2003 outbreak of this coral disease, called acroporid serratiosis (APS), a unique strain of the pathogen, *Serratia marcescens* strain PDR60, was identified from diseased *A. palmata*, human wastewater, the non-host coral *Siderastrea siderea* and the corallivorous snail *Coralliophila abbreviata*. In order to examine humans as a source and other marine invertebrates as vectors and/or reservoirs of the APS pathogen, challenge experiments were conducted with *A. palmata* maintained in closed aquaria to determine infectivity of strain PDR60 from reef and wastewater sources. Strain PDR60 from wastewater and diseased *A. palmata* caused disease signs in elkhorn coral in as little as four and five days, respectively, demonstrating that wastewater is a definitive source of APS and identifying human strain PDR60 as a coral pathogen through fulfillment of Koch's postulates. *A. palmata* inoculated with strain PDR60 from *C. abbreviata* showed limited virulence, with one of three inoculated fragments developing APS signs within 13 days. Strain PDR60 from non-host coral *S. siderea* showed a delayed pathogenic effect, with disease signs developing within an average of 20 days. These results suggest that *C. abbreviata* and non-host corals may function as reservoirs or vectors of the APS pathogen. Our results provide the first example of a marine “reverse zoonosis” involving the transmission of a human pathogen (*S. marcescens*) to a marine invertebrate (*A. palmata*). These findings underscore the interaction between public health practices and environmental health indices such as coral reef survival.

## Introduction

**Figure 1:**
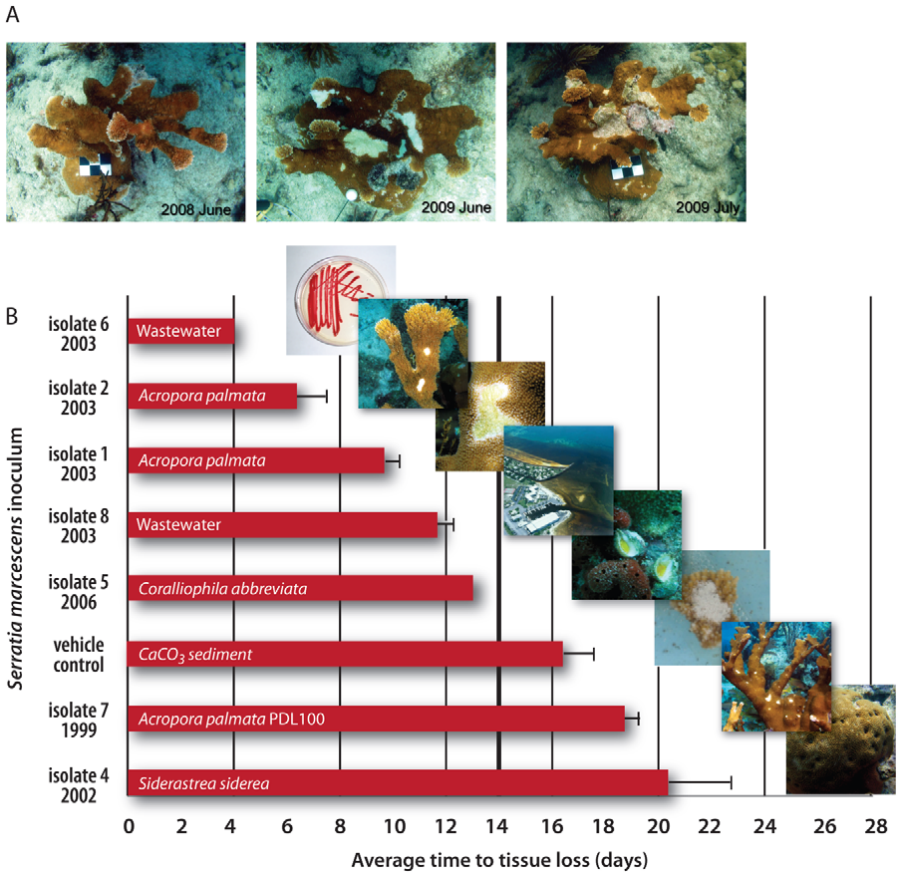
Time series of white pox affected *Acropora palmata* at Looe Key Reef in the Florida Keys and average time to tissue loss in the challenge experiments. A. Images of white pox affected *A. palmata* at Looe Key, from June 2008, to June 2009, to July 2009 (left to right), show colony growth and partial mortality. Scales bars are 3 cm on a side or 3 cm in diameter. (Photographs by JW Porter and MK Meyers) B. Average time (days) to development of disease signs on *A. palmata* inoculated with *Serratia marcescens*. Days to tissue loss was averaged for the three *A. palmata* fragments used in each treatment and each control. The original inoculum was recovered for all seven of the presented *S. marcescens* inocula, including five isolates of strain PDR60 collected from acroporid serratiosis (APS)-affected *A. palmata* (isolates 1, 2), non-host coral *Siderastrea siderea* (isolate 4), corallivorous snail *Coralliophila abbreviata* (isolate 5), and untreated wastewater (isolate 6). Two additional inocula included *S. marcescens* strain PDL100 (isolate 7), previously confirmed as an APS pathogen through fulfillment of Koch's Postulates (2) and *S. marcescens* strain WWI31 from untreated wastewater (isolate 8). The vehicle control exhibited tissue loss beginning at day 15. The *E. coli*-plus-vehicle control and the isolate 3-plus-vehicle treatment did not exhibit tissue loss and remained apparently healthy for the duration of the 26 day study. Virulent strains (isolates 1, 2, 5, 6, 8) caused tissue loss within 14 days of inoculation and attenuated strains (isolates 4, 7) caused tissue loss after day 14.

Once the most common coral in the Caribbean, elkhorn coral *Acropora palmata* was listed for protection under the United States Endangered Species Act in 2006 [1], largely due to a disease unique to this coral species (Fig. 1). This disease, termed white pox or acroporid serratiosis (APS), is caused by the bacterium *Serratia marcescens* [2]. *S. marcescens* is an opportunistic human pathogen, causing respiratory, wound and urinary tract infections, meningitis and pneumonia [3]–[10]. Human diseases caused by this bacterium have been associated with waterborne infections in tropical freshwaters [11], but are most often associated with nosocomial infections of neonates and immuno-compromised patients [12]. During a 2003 outbreak, a unique strain of *S. marcescens*, strain PDR60, was identified from both diseased *A. palmata* and untreated human sewage, suggesting a causal link between wastewater and APS [13]. In addition, strain PDR60 was isolated from potential vectors and reservoirs including the non-host corals *Siderastrea siderea* and *Solenastrea bournoni* and the coral predatory snail *Coralliophila abbreviata* [13].

Koch's postulates, the preferred method for proving disease causation in humans and wildlife, require inoculation of a pure culture of a suspected pathogen isolated from a diseased host onto an apparently healthy host followed by development of disease signs and reisolation of the pathogen. Koch's postulates have proven difficult to fulfill for a number of coral diseases due in part to difficulty isolating and identifying potential pathogens and growing these organisms in pure culture. To date, the postulates have been satisfied for only five coral diseases, including APS with strain PDL100 isolated from a 1999 outbreak [2], [14]. Unlike strain PDR60, isolated from host and non-host corals and snails, strain PDL100 has been identified only from diseased *Acropora palmata* [13]. Here we hypothesize that strain PDR60 isolated from two distinct environments, one terrestrial (human wastewater) and one marine (APS-affected *A. palmata*, apparently healthy *Siderastrea siderea* and *Coralliophila abbreviata*) causes APS in *A. palmata*. To examine this hypothesis we conducted challenge experiments by inoculating eight isolates of *Serratia marcescens* representing three strains onto *A. palmata* fragments maintained in closed seawater aquaria. Our results confirm strain PDR60 as a coral pathogen through fulfillment of Koch's postulates. These results are also consistent with the hypothesis that non-host corals and predatory snails may function as interepizootic reservoirs or vectors of the APS pathogen. Furthermore, we show that *S. marcescens* isolated from human wastewater causes APS in as little as four days, unequivocally verifying humans as a source of a marine invertebrate disease.

## Materials and Methods

### Challenge Experiment Design

Experiments were conducted in June 2009 at the Mote Tropical Research Laboratory (MTRL), Summerland Key, FL. Eleven aquaria (20 L) were placed in a wet-table raceway with flowing 20 µm filtered groundwater and filled with 10 µm filtered seawater. A pump was placed in each aquarium, approximately 1 cm below the water surface, to maintain aeration. The raceway was covered with a plastic tarp and this shading plus water circulating around aquaria maintained aquaria temperatures at 28°C. Aquaria containing seawater acclimated for 24 hours before corals were added.

Small *Acropora palmata* fragments (25 cm^2^) were collected from 19 apparently healthy colonies at Western Sambo Reef, FL. Prior to fragment collection, the surface mucopolysaccharide layer (SML) was removed from each of the 19 coral colonies using sterile needle-less 20 cm^3^ syringes and placed in a cooler on ice. Coral fragments were placed in 1 gallon zip-lock plastic bags containing seawater and stored in a cooler without ice. SML and coral samples were transported to the MTRL. Within three hours of collection, SML samples were spread plated (100 µl) on MacConkey Sorbitol agar (Becton Dickinson, Franklin Lakes, NJ, USA) with colistin (200 U ml-1; MP Biomedicals, Aurora, OH, USA) (MCSA) [15]. Inoculated MCSA plates were incubated overnight at 37°C.

The number of coral fragments collected per coral colony ranged from one to four (Table 1). Within three hours of collection, these fragments were distributed into aquaria, three fragments per aquarium, with the goal of limiting replication of fragments from any one colony in a single aquarium. For colonies from which more than one fragment was collected, this distribution of fragments allowed for genotype comparisons across treatments (Table 1). Clonal replication within an aquarium was measured in aquariums 4 and 10 (Table 2) to determine genotype response to a single treatment. Coral fragments acclimated in seawater aquaria for 48 hours prior to commencement of inoculation experiments.

**Table 1:** *Acropora palmata* colonies used in the challenge experiments.

Colony	Number of Fragments	Tissue Loss	No Tissue Loss
1	2	F1 Isolate 2 *A. palmata*F1 Isolate 8 wastewater	
2	1	F3 Isolate 4 *S. siderea*	
3	1		F1 Isolate 5 *C. abbreviata*
4	3	F3 Isolate 2 *A. palmata*F1 Isolate 7 PDL100F3 Isolate 7 PDL100	
5	3	F2 Isolate 4 *S. siderea*F2 Isolate 6 wastewater	F3 seawater control
6	2	F3 vehicle control	F3 Isolate 3 *A. palmata*
7	4	F1 Isolate 1 *A. palmata*F2 Isolate 1 *A. palmata*F2 Isolate 2 *A. palmata*F1 Isolate 4 *S. siderea*	
8	1		F1 seawater control
9	1	F1 vehicle control	
10	2	F3 Isolate 5 *C. abbreviata*F2 Isolate 7 PDL100	
11	2	F3 Isolate 8 wastewater	F2 *E. coli* control
12	1	F1 Isolate 6 wastewater	
13	3		F1 Isolate 3 *A. palmata* F2 Isolate 5 *C. abbreviata*F3 *E. coli* control
14	2	F3 Isolate 1 *A. palmata*F2 Isolate 8 wastewater	
15	1	F2 vehicle control	
16	1		F1 *E. coli* control
17	1		F2 Isolate 3 *A. palmata*
18	1	F3 Isolate 6 wastewater	
19	1		F2 seawater control

Number of fragments per *A. palmata* colony, the challenge for each fragment (control or inoculation with a *Serratia marcescens* test isolate), and the result of each challenge (tissue loss or no tissue loss) are included. Three coral fragments (F1, F2, F3) were used for each challenge.

**Table 2:** Treatments and controls used in the challenge experiments.

Aquarium	Coral Fragment	Coral Colony	Challenge	No. Days To Tissue Loss
1	F1	8	Seawater Control	No Tissue Loss
	F2	19		No Tissue Loss
	F3	5		No Tissue Loss
2	F1	16	*E.coli* Control	No Tissue Loss
	F2	11		No Tissue Loss
	F3	13		No Tissue Loss
3	F1	9	Vehicle Control	17
	F2	15		15
	F3	6		17
4	F1	7	Isolate 1 *Acropora palmata*	10
	F2	7		10
	F3	14		9
5	F1	1	Isolate 2 *A. palmata*	7
	F2	7		5
	F3	4		7
6	F1	13	Isolate 3 *A. palmata*	No Tissue Loss
	F2	17		No Tissue Loss
	F3	6		No Tissue Loss
7	F1	7	Isolate 4 *Siderastrea siderea*	19
	F2	5		23
	F3	2		19
8	F1	3	Isolate 5 *Coralliophila abbreviata*	No Tissue Loss
	F2	13		No Tissue Loss
	F3	10		13
9	F1	12	Isolate 6 Wastewater	4
	F2	5		4
	F3	18		4
10	F1	4	Isolate 7 PDL100 *A. palmata*	19
	F2	10		18
	F3	4		19
11	F1	1	Isolate 8 Wastewater	11
	F2	14		12
	F3	11		12

A total of eleven challenge experiments were conducted each within a single aquarium and each with three *Acropora palmata* coral fragments (F1, F2, F3). Coral colony of origin and number of days to tissue loss is included for each coral fragment. Treatments and controls for which there was no tissue loss are also noted.

### Challenge Experiment Inoculations

Test isolates used in the challenge experiments were collected between June 2002 and August 2006 from off-shore coral reefs and wastewater in the Florida Keys (Table 3). These *Serratia marcescens* isolates were cultured and identified from environmental samples by Sutherland *et al.* [13] using a three-step method: culture on MCSA followed by culture on DNase with Toluidine Blue agar (DTC; Becton Dickinson) amended with cephalothin (0.1 mg ml^−1^; ICN Biomedicals, Aurora, OH, USA) [16] followed by *Serratia*-specific PCR with primers Smar 16SV (GGGAGCTTGCTCACTGGGTG) and Smar 16SWR (GCGAGTAACGTCAGTTGATGAGCGTATTA) (Sigma-Genosys, Woodlands, TX) [17]. The efficiency of these methods for the isolation of *S. marcescens* was confirmed with sequencing of 16S rDNA PCR amplicons [13]. Genotypic fingerprinting (by endonuclease restriction) of environmental isolates of *S. marcescens* was conducted with pulsed-field gel electrophoresis (PFGE), based on the protocols of Miranda *et al.* [8] and Shi *et al.* [9], in order to determine relatedness between isolates and to assess genetic similarity among and between strains [13]. PFGE is widely recognized as an outstanding method for molecular strain typing of *S. marcescens* and is commonly employed to source track clinical outbreaks caused by this bacterium [8], [9], [18]–[25].

**Table 3:** Bacterial treatments inoculated onto the *Acropora palmata* fragments in the challenge experiments.

Isolate	Strain	Source of Collection	Location of Collection	Date of Collection
1	PDR60	APS-affected *Acropora palmata* coral	Rock Key	July 2003
2	PDR60	APS-affected *Acropora palmata* coral	Grecian Rocks	July 2003
3	PDR60	APS-affected *Acropora palmata* coral	Grecian Rocks	July 2003
4	PDR60	*Siderastrea siderea* coral	Long Key	June 2002
5	PDR60	*Coralliophila abbreviata* snail	Sand Key	August 2006
6	PDR60	wastewater	Key West	September 2003
7	PDL100	APS-affected *Acropora palmata* coral	Looe Key	May 1999
8	WWI31	wastewater	Key West	September 2003

Eight isolates of *Serratia marcescens* representing three pulsed-field gel electrophoresis (PFGE) strains (PDR60, PDL100, and WWI31) were used. The source, location, and date of collection are included for each treatment.

Six of the test isolates used in the challenge experiments were a single PFGE-identified strain, *Serratia marcescens* PDR60, collected from APS-affected *Acropora palmata* at Rock Key reef in the lower Keys (isolate 1) and Grecian Rocks reef in the upper Keys (isolates 2, 3), the apparently healthy non-host coral *Siderastrea siderea* (isolate 4) at Long Key, the corallivorous snail *Coralliophila abbreviata* (isolate 5) at Sand Key reef, and from untreated wastewater (isolate 6) from the Key West wastewater treatment facility (Table 3) [13]. The known coral pathogenic strain, *S. marcescens* PDL100 (isolate 7) [2] and one additional isolate, representing a different *S. marcescens* strain, WWI31, from untreated wastewater (isolate 8) [13] were also tested (Table 3). Controls included *Escherichia coli* (ATCC 15597), vehicle (sterile CaCO_3_ sediment), and seawater. One aquarium containing three coral fragments was used for each treatment and each control (Table 2). Due to the threatened status of *A. palmata*, the scope of our study was limited to eight treatments and three controls, each in a single dose, by the small size and low number of coral fragments that were permitted for collection.

Bacterial cultures of test isolates were prepared by combining 15 ml trypticase soy (TS) broth (Becton Dickinson, Sparks, MD, USA) and 15 g sterile sieved (1 mm internal diameter) CaCO_3_ sediment in sterile 50 ml tubes and inoculating each broth-sediment mixture with a test isolate. Vehicle control was prepared as above, but without bacterial inoculum. Inoculated mixtures were incubated overnight (25°C) with horizontal shaking and grown to 10^7^ colony forming units ml^−1^. Overnight cultures were centrifuged (5 min, 1000× g) and supernatant discarded. Sterile artificial seawater (ASW, 15 ml) was added to the pellet containing cells and sediment and mixed by inversion. Inoculations were performed by depositing 0.85 g of each bacterial absorbed sediment mixture directly onto triplicate fragments in a single aquarium. Fragments that did not develop disease signs within seven days (Table 2) were inoculated again with fresh bacterial absorbed sediment. Fragments were photographed immediately before and after inoculation.

### Data Collection

The number of days to tissue loss for each coral fragment was calculated from the first experimental inoculation (Table 2). When tissue loss was observed, the fragment was photographed and SML (containing necrotic coral tissue) was collected from the lesion margin using a sterile needle-less 20 cm^3^ syringe. SML was transferred to a sterile 15 ml plastic tube and centrifuged (5 min, 1000× g) and supernatant discarded. The pellet was resuspended in ASW (500 ml) and spread plated (100 µl) in triplicate on MCSA. Inoculated MCSA plates were incubated overnight at 37°C. MCSA positive bacterial colonies, appearing pink to red on MCSA (characteristic of *Serratia marcescens*), were picked and plated onto DTC. Inoculated DTC plates were incubated overnight at 41°C. Presumptive *S. marcescens*, determined by a DNase-positive reaction (appearance of a red halo on DTC), were streaked onto non-selective TS agar and incubated overnight at 25°C for confirmation of pure colonies. Experiments were monitored several times daily. Partial water changes were conducted at least once daily and salinity was maintained at 35 (measured by refractometry). After 26 days experiments were concluded. All fragments remaining alive were apparently healthy. Each fragment was photographed and then SML was collected and processed as described above. At the conclusion of experiments, or after development of disease signs and examination of SML, each *Acropora palmata* fragment was fixed with Z-Fix concentrate (ANATECH, LTD, Battle Creek, MI, USA) diluted 1∶4 with ASW and stored for future study.

### Strain Typing of APS Pathogens

Isolates recovered from APS-affected *Acropora palmata* fragments in inoculation experiments and identified as presumptive *Serratia marcescens* by differential growth on MCSA and DTC were assayed with *Serratia*-specific PCR as described above and in Sutherland *et al.* [13] to confirm the identity of isolates as *S. marcescens*. PCR-confirmed *S. marcescens* were assayed with PFGE as described in Sutherland *et al.* [13]. Band patterns were used to determine relatedness between isolates in order to confirm that bacteria collected from APS lesions produced during inoculation experiments were the same strain of bacterium inoculated in each inoculation experiment, thereby fulfilling Koch's postulates.

## Results

*Serratia marcescens* were not identified from SML of any *Acropora palmata* fragments prior to use in challenge experiments or from any apparently healthy fragments remaining alive at the end of the study. Seawater circulating around aquaria, seawater used for water changes in aquaria, and deionized water used to adjust salinity were all negative for *S. marcescens*. *A. palmata* fragments in the *E. coli*-plus-vehicle control, the seawater-alone control, and the isolate 3-plus-vehicle treatment aquaria remained apparently healthy and were maintained for the duration of the 26 day study. In the vehicle control aquarium, coral tissue loss was noted by day 15 (Table 2). Therefore, test inocula were considered virulent if disease signs occurred prior to day 15, or within 14 days of the first inoculation, and attenuated if disease signs developed after 14 days.

When disease occurred, the process began with the distinct smell of *Acropora palmata* mucus followed by decreased water clarity, presumably due to the release of mucus by the coral into the water. Seawater in the affected aquaria was changed every two to five hours once these signs developed. Mucus release was followed by a tissue bleb adjacent the region of inoculation. Finally, the tissue lifted off the skeleton and was sloughed in stringy fragments. This process of disease progression, once begun, was completed within 24 hours.

Koch's postulates were satisfied for *Serratia marcescens* strain PDR60 from APS-affected corals (isolates 1, 2). *Acropora palmata* fragments also developed APS signs when inoculated with two *S. marcescens* strains from wastewater [strain PDR60 (isolate 6) and strain WWI31 (isolate 8)] and with strain PDR60 from healthy non-host corals (isolate 4) and snails (isolate 5); however, individual strains showed varying degrees of pathogenic effect. Strain PDR60 from APS-affected corals and wastewater (isolates 1, 2, 6) caused disease signs within four to ten days (Table 2, Fig. 1). Strain WWI31 (isolate 8) from wastewater caused disease signs within 12 days. Isolate 4 (strain PDR60 from *Siderastrea siderea*) and strain PDL100 (isolate 7), were attenuated, and only caused disease signs at 23 and 19 days, respectively (Table 2, Fig. 1). PFGE analyses demonstrated that the original inocula were recovered from all APS lesions.

## Discussion

*Serratia marcescens* strain PDR60 isolated from wastewater and diseased *Acropora palmata* caused APS in as little as four days, confirming humans as a source of this disease. Although the scope of this study was limited to three replicate *A. palmata* fragments in a single aquarium for each treatment and each control, our results with strain PDR60 from wastewater, predatory snails, and non-host corals contribute to the understanding of the mechanisms of transmission of APS and of the factors that drive the emergence and maintenance of this marine epizootic.

*Acropora palmata* inoculated with strain PDR60 from the non-host coral *Siderastrea siderea* (isolate 4) and strain PDL100 from APS-affected host corals (isolate 7) showed an attenuated pathogenic affect (Fig. 1). These two isolates caused APS signs after the fragments in the vehicle control showed tissue loss. All other treatments that exhibited tissue loss, did so by day 13, two days before the vehicle control (Table 2). For this reason, results for isolates 4 and 7, may indicate that stressors other than *Serratia marcescens* infection contributed to tissue loss; however, the maintenance of the *E. coli*-plus-vehicle control and the isolate 3-plus-vehicle treatment for the duration of the 26 day study lends support to the pathogenic effects of isolate 4 and isolate 7. Results with isolate 4 indicate that *S. siderea*, and possibly other non-host corals (*e.g.*, *Solenastrea bournoni*) [13], may serve as a reservoir for the acroporid pathogen in the reef environment. Previous work suggests that PDL100 is poorly adapted for survival in the marine environment, persisting in seawater for only 15 h [26], but survival and proliferation of this strain is enhanced when it is grown in *A. palmata* SML and *S. siderea* SML [26], [27]. Our results indicate that PDL100 survived for at least 11 days in association with *A. palmata*; PDL100 was recovered from an APS lesion on day 19 of the experiment (Table 2), 11 days after the second experimental inoculation. *S. marcescens* resident in non-host SML may be transmitted to neighboring *A. palmata* by sloughing of SML or movement by *Coralliophila abbreviata* vectors [13].

*Coralliophila abbreviata* preys preferentially on *Acropora palmata* [28] and has been implicated in the transmission of an unknown disease of *Acropora cervicornis* [29]. Strain PDR60 from *C. abbreviata* (isolate 5) was virulent, but only in one of three fragments (Table 2). Colony 13, a colony for which genotype comparisons across treatments were conducted, did not develop APS signs when inoculated with isolate 5 (Table 2) or with any other treatment (Table 1). The limited virulence of isolate 5 may be due to the apparent APS resistance of colony 13 or to the specific origin of this isolate, collected in 2006 at Sand Key reef when no sign of white pox was apparent at that reef [13]. These experimental and field observations support the limited virulence of isolate 5, but virulence of this isolate against one *A. palmata* fragment is consistent with the hypothesis that *C. abbreviata* may function as an interepizootic reservoir of the APS pathogen or as a vector when snails that harbor the pathogen feed on host corals.

*Acropora palmata* inoculated with isolate 3 from APS-affected corals remained apparently healthy for the entirety of the experiment, indicating that either this isolate of strain PDR60 is non-virulent or that the corals on to which it was inoculated were resistant to APS. Evidence in support of the former conclusion is that the three *A. palmata* fragments inoculated with isolate 3 were each a different genotype and all remained apparently healthy for the duration of the 26 day study (Table 2). The later conclusion is supported by the apparent APS resistance of colony 13 (Table 1). Observations of *A. palmata* populations suggest that colonies of this species that are resistant to APS may exist. Prevalence of white pox disease has declined in recent years coincident with declines in host coral populations. The most recent reported outbreaks of the disease occurred in the Florida Keys in 2003 [13] and 2009 (Fig. 1) and in St. John, US Virgin Islands in 2006 [30]. Lower disease prevalence may be due to the decimation of susceptible *A. palmata* populations during previous white pox epizootics [2], [31], other disease outbreaks [32], [33], or may be an indication of reduced pathogen virulence [34].

*Serratia marcescens* strain PDL100 exhibited attenuated virulence in this study, and this strain's reduced pathogenic effect, combined with the virulence of strain PDR60 and the wastewater strain WWI31, raises the question of whether unique strains exist for other coral pathogens. For instance, coral disease pathogens that cause white plague (*Aurantimonas corallicida*) and bacterial-induced bleaching (*Vibrio shiloi*) have waned in pathogenicity in recent years [34]. Limited pathogenicity of *A. corallicida*, *V. shiloi*, and *S. marcescens* PDL100 may indicate the evolution of host-resistance to these pathogens [34].

We have shown that distinct strains of *Serratia marcescens* are capable of causing disease in *Acropora palmata* and that strains show varying levels of apparent virulence with time to disease lesions ranging from 4 days to 23 days (Table 2). These results may explain variability in manifestation of APS lesions on *A. palmata* colonies in the field including time to infection, rate of tissue loss, and likelihood of recovery versus whole-colony death. Future studies will investigate host susceptibility and resistance and will evaluate genetic variation between *S. marcescens* isolates and strains confirmed to be pathogenic and those that do not cause disease or cause disease at a slower rate. There is a need to define key differences between *S. marcescens* strains (PDL100 and PDR60) as well as among isolates of the same strain (PDR60, *e.g.*, isolates 1–6). Important differences may exist in their ability to adapt to marine conditions (*i.e.*, ability to persist and/or grow in seawater) and in genetic differences leading to increased virulence. *S. marcescens* vary widely in their survival in seawater with strain PDL100 surviving for 15 h or less at 30°C [26] and marine-derived isolates of the PDR60 pathogen strain persisting for up to 20 days under the same conditions (KP Sutherland and EK Lipp unpublished data). Fitness of different *S. marcescens* strains in seawater may also shed light on the mode of APS transmission. For example, saltwater tolerant strains may be more likely to become persistent or even resident on the reef whereas less halo-tolerant strains may either die off before reaching the reef or may be responsible for discrete outbreaks over short time periods. Our data from microcosm studies suggest that PDL100 (isolated in 1999) follows the latter pattern [26] and is supported by the fact that this strain has never been re-isolated from the marine environment [13]. On the other hand, strain PDR60, which has been recovered from multiple marine sources [13], is highly persistent in marine waters (KP Sutherland and EK Lipp unpublished data), suggesting that this may be an endemic disease strain.

Human culpability in the demise of a threatened species necessitates an immediate response and supports ongoing mitigation to improve wastewater treatment in the Florida Keys, and elsewhere, in order to protect the health and biodiversity of coral reef ecosystems. Advanced wastewater treatment successfully removes *Serratia marcescens* to undetectable levels [13]. However, most wastewater in the Keys and wider Caribbean is not treated, but rather is disposed of through in-ground receptacles [35] within porous limestone substrate that permits leakage from these systems into near-shore waters [36]. Human fecal contamination of near-shore and off-shore coral reef environments has been clearly demonstrated in the Florida Keys [35]–[38] and elsewhere in the Caribbean [39] and is associated with waterborne disease in humans [40]. In response, the state of Florida passed legislation to improve water quality in the Florida Keys by requiring the upgrade of all wastewater facilities, including in-ground receptacles, to the best available technology or to advanced wastewater treatment at an estimated cost of $939 million [41].

Coral reefs are amongst the most critically endangered habitats on earth. Because population declines of *Acropora palmata* are caused, in part, by a human strain of the common fecal enteric bacterium, *Serratia marcescens*, our findings address not only environmental protection, but also the socioeconomic and socio-ecological determinants of coastal zone protection and the cost of wastewater treatment infrastructure. This study brings to light a disease system dynamic, from humans to wildlife, which is the opposite of the traditional wildlife-to-human disease transmission model. The passage of pathogens from wildlife to humans is well documented, but the movement of disease-causing microbes from humans to marine invertebrates has not been shown [42]. This “reverse zoonosis” is all the more interesting because it involves the jump of a pathogen from vertebrate to invertebrate and from terrestrial to marine. Furthermore, disease incidence or severity may increase with rising temperatures [26], [43], [44], reinforcing the importance of near-shore water quality under climate change scenarios. Given a reliance on tourism by most Caribbean countries and the widespread lack of consistent wastewater treatment in the region, the APS disease system is of great economic interest and public health concern to developing nations and has particular significance for sustainable development activities and coastal-zone carrying capacity studies world-wide, especially under changing climatic conditions.
